# Monitoring EML4-ALK Biomarkers via a Microfluidic Assay for Treatment Efficacy Assessment

**DOI:** 10.3390/bios16090484

**Published:** 2026-09-02

**Authors:** Shaked Doron, Lev Brio, Matan Krasner, Efrat Barbiro-Michaely, Doron Gerber

**Affiliations:** The Mina and Everard Goodman Faculty of Life Sciences and the Institute for Nanotechnology and Advanced Materials, Bar Ilan University, Ramat-Gan 5290002, Israel

**Keywords:** integrated microfluidics, minimal residual disease, NSCLC, anaplastic lymphoma kinase, CTCs

## Abstract

Monitoring prognostic biomarkers is essential for evaluating cancer treatment efficacy in real time. Here, we present a rapid, proof-of-concept microfluidic assay for cancer biomarker quantification and functional therapy evaluation. The assay requires only 5 µL of sample, eliminates tedious sample manipulation, and uses commercial antibodies to track both biomarker concentration and functional activity. We demonstrated the successful detection of VEGF and EML4-ALK proteins in both lung cancer cell culture supernatant and serum-spiked samples, achieving a detection sensitivity 15 times greater than standard ELISA using the same antibody pairs for the respective antigens. Crucially, we showcase the platform’s distinct applicability to functional assays by monitoring dynamic phosphorylation changes in EML4-ALK following treatment with the tyrosine kinase inhibitor alectinib, successfully capturing a significant, rapid decrease in phosphorylation levels. At this preclinical stage, the platform demonstrates analytical and functional feasibility for highly sensitive and rapid biomarker monitoring, providing a foundation for future validation in patient-derived ALK-positive samples for therapeutic-response assessment.

## 1. Introduction

The real-time evaluation of therapeutic response represents a critical paradigm shift in contemporary oncology, transitioning the clinical management landscape from static diagnostic classifications to active, dynamic therapeutic monitoring [1,2]. While liquid biopsy platforms offer an unprecedented non-invasive window for tracking disease status across diverse treatment modalities [3], their routine translation into hospital workflows is heavily bottlenecked by the analytical sensitivity, reproducibility, and turnaround limits of current profiling technologies [4,5]. Next-generation sequencing (NGS) stands as the undisputed gold standard for initial, comprehensive genomic mapping; however, its clinical utility in guiding agile, real-time therapy adjustments is significantly limited by high operational costs, lengthy processing timelines [6], and a structural inability to capture transient, post-translational protein behavior or instantaneous functional enzymatic states under drug pressure [7]. Conversely, conventional protein macro-assays, predominantly enzyme-linked immunosorbent assays (ELISAs), are severely constrained by high sample volume requirements, labor-intensive workflows, and a lack of point-of-care portability, rendering them incapable of tracking rapid biomarker kinetics from sparse clinical volumes [8].

To overcome these analytical bottlenecks, a multi-modal diagnostic strategy that concurrently captures intracellular oncogenic drivers and extracellular microenvironmental regulators offers profound clinical utility. A classic paradigm of an intracellular driver mutation is the Echinoderm Microtubule-associated protein-like 4-Anaplastic Lymphoma Kinase (EML4-ALK) fusion protein, arising from a recurrent paracentric inversion on chromosome 2p [9,10]. This genetic rearrangement results in constitutive, ligand-independent tyrosine kinase domain activation, driving uncontrolled survival and proliferation signals in 3% to 5% of non-small-cell lung cancer (NSCLC) patients [11], and belonging to a broader family of targetable fusions (including ROS1, RET, and NTRK1/2/3) that act as clonal drivers in up to 10% of lung adenocarcinomas [12]. Coordinated alongside this intracellular axis, Vascular Endothelial Growth Factor (VEGF) acts as a fundamental extracellular microenvironmental regulator secreted under hypoxia to orchestrate tumor angiogenesis, nutrition, and metastatic progression [13]. Upregulated circulating levels of VEGF are highly prevalent across multiple solid tumors [14] and serve as established indicators of therapeutic resistance and poor survival outcomes [15]. Crucially, both biomarkers are actively packaged and secreted within tumor-derived extracellular vesicles, or exosomes; exosomal cargo encapsulation shields functional EML4-ALK proteins from enzymatic degradation [16], while exosomal VEGF isoforms directly stimulate endothelial cell receptors to drive aggressive neo-vascularization [17]. Consequently, tracking this dual axis from a unified, unpurified liquid biopsy represents a highly comprehensive approach to monitoring the evolving disease state.

The clinical management of patients harboring these targetable fusions relies heavily on small-molecule tyrosine kinase inhibitors (TKIs), with the highly selective agent alectinib establishing the current first-line standard of care for EML4-ALK-positive NSCLC [18]. Alectinib competitively blocks the adenosine triphosphate (ATP)-binding pocket of the ALK kinase domain, successfully arresting downstream oncogenic signaling cascades and halting cellular proliferation [19,20]. While these targeted interventions are prescribed based on static genetic verification, static genomic profiles fail to reflect real-time, post-translational kinase activity or immediate functional shifts under pharmacological challenge, creating a dangerous diagnostic latency window [21]. Clinicians currently lack tools for rapid validation and must wait several months to evaluate therapeutic efficacy via macro-scale radiographic updates and standard Response Evaluation Criteria in Solid Tumors (RECIST). This latency highlights an urgent need for functional, kinetic assays capable of mapping immediate target phosphorylation changes directly under treatment pressure within hours of biopsy collection, offering a predictive window into patient-specific drug sensitivity or emerging resistance.

Microfluidics and lab-on-a-chip technologies represent a powerful paradigm to bridge this clinical gap by enabling rapid, decentralized, and ultra-sensitive molecular diagnostics [4,22]. By manipulating sub-microliter fluid volumes within precisely patterned microchannels, microfluidic platforms exploit predictable laminar flow regimes to deliver accelerated reaction kinetics, highly minimized reagent consumption, and drastically reduced sample volume requirements [23]. Despite these extensive operational benefits, widespread translation of existing microfluidic biosensors has been structurally hindered by their reliance on complex, multi-step surface functionalization chemistry, specialized cleanroom nanofabrication, or custom-synthesized capture agents. To achieve authentic routine utility, clinical monitoring assays must integrate seamlessly into standard hospital workflows without extensive pre-analytical processing, pairing robust microfluidic architectures with flexible surface chemistry protocols that utilize commercially available, off-the-shelf antibodies [24].

In this study, we present a rapid, preclinical proof-of-concept integrated microfluidic platform designed for simultaneous concentration, physical quantification, and functional therapy profiling of EML4-ALK and VEGF directly within complex, unpurified clinical matrices. The microfluidic assay requires a sample volume of only 5 µL, eliminates tedious off-chip pre-analytical sample manipulation or exosome isolation steps, and utilizes flexible surface chemistry protocols integrated with commercial, off-the-shelf antibodies. We demonstrate successful target resolution in both lung cancer cell culture supernatants and crude serum-spiked samples, achieving an analytical detection sensitivity superior to the corresponding conventional plate-based ELISA using identical antibody pairs. Crucially, we demonstrate the unique utility of our platform for functional oncology by capturing the rapid, dynamic decreases in EML4-ALK tyrosine phosphorylation levels following targeted treatment with the TKI alectinib, demonstrating an outstanding physiological correlation with established cell-free IC50 values. This robust, automated platform enables highly sensitive, specific, and rapid monitoring of therapeutic response, supporting its future development as a clinical tool for therapy-response monitoring, pending validation in prospectively collected patient samples.

## 2. Results

**Figure 1:**
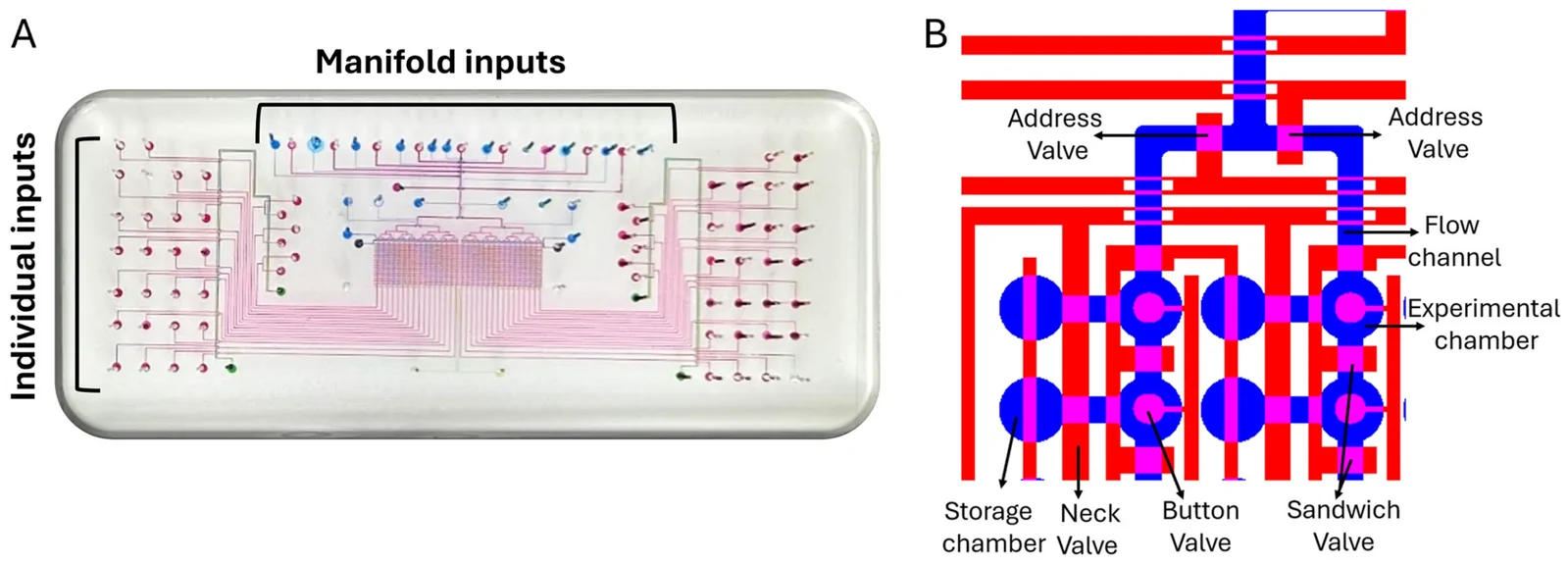
Schematic presentation of the microfluidic device used for on-chip detection and quantification of EML4-ALK protein. (**A**) The integrated microfluidic device builds from two layers. The flow tubes layer (blue) serves for all experimental reagents application, and the control layer (red) controls reagents flow within the device. (**B**) Closer view into the device presents the alignment between both layers. The control layer possesses three types of valves; the neck valve—separates the experimental chamber from the storage chamber, isolating the experimental surroundings; the sandwich valve—allows isolation of the experimental chamber from its surroundings, allowing 32 repetitions for each column; the button valve—is used for mechanical trapping of the ELISA assay beneath it and allows washing rounds through the entire experiment with no risk of signal loss.

In the present study, we designed and manufactured a microfluidic device (Figure 1) that utilizes a quick two-step protocol for the immobilization and detection of different protein biomarkers [25] using off-the-shelf antibodies, widely used for ELISA experiments, as well as familiar proteins in the field of cancer, such as biomarker candidates. Protocol calibration experiments lead us to choose a model in which the sample is flowed through the microfluidic device allowing maximal accumulation of the molecule of interest (by the anchoring antibody) and with high detection sensitivity, which is mostly desired in cases where the biomarker concentration within the sample is low (Figure S1).

**Figure 2:**
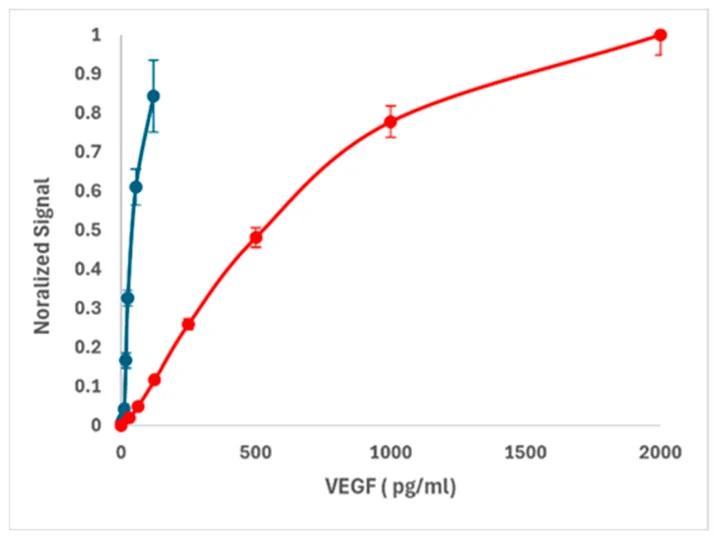
Common ELISA vs. on-chip ELISA using reagents from a commercial ELISA kit. A dose–response experiment of VEGF ELISA on a chip (blue) versus standard ELISA (red) using the same antibodies and reagents. Data represents 3 independent experiments, normalized between maximum and background signals, with a total of N = 30 repeats.

The first biomarker chosen was VEGF, a known biomarker involved in many forms of cancer and a popular therapeutic target. There are many commercially available antibodies and kits for VEGF detection [26,27]. We tested the Human VEGF DuoSet ELISA kit (Catalog # DY293B) for its detection threshold in regular ELISA compared to the microfluidic platform (Figure 2). A comprehensive statistical analysis was performed (Table 1). We report; limit of detection LOD concentration, fitting slope (S) at the linear zone at the lower protein concentrations, limit of quantitative detection (LOQ) and precision/CV%. Dose–response were mathematically fitted using a standard 4-parameter logistic curve yielding a regression value of R^2^ > 0.99. All equations are presented in the Methods (Section 4.7). The blank distribution, in the on-chip experiment, was established by measuring n = 30 independent baseline zones from three independent experiments, yielding a mean intensity of 0.0055 A.U. with a standard deviation of 0.00431 A.U. Notably, the on-chip sensitivity was 15 times better than the corresponding standard ELISA and the CV% was eight times smaller at the lowest concentration. Analytical sensitivity was also evaluated by the curve slope at the linear zone. The shallow slope of the standard ELISA (0.00257) means that a lot of protein is needed to clearly overcome the background noise and induce an increase in the signal, whereas the on-chip slope (0.0125) is roughly five times steeper than the standard ELISA slope meaning that even small amounts of protein change the signal. This significant difference points out the higher sensitivity of on-chip ELISA versus the standard ELISA.

Next, we turned to the detection of EML4-ALK, a much more challenging cancer marker that cannot be detected in blood samples using a common ELISA. On average, human blood contains 80 mg/mL of total protein, and isolating or detecting a target protein from this “soup” is highly challenging [28]. It usually involves protein purification or serum dilution, both of which decrease assay sensitivity and require a large volume of blood. We confirmed this using Western blotting to simulate the standard detection of EML4-ALK with antibodies, where our lowest detectable concentration was 5 ng/mL (Figure S3).

**Figure 3:**
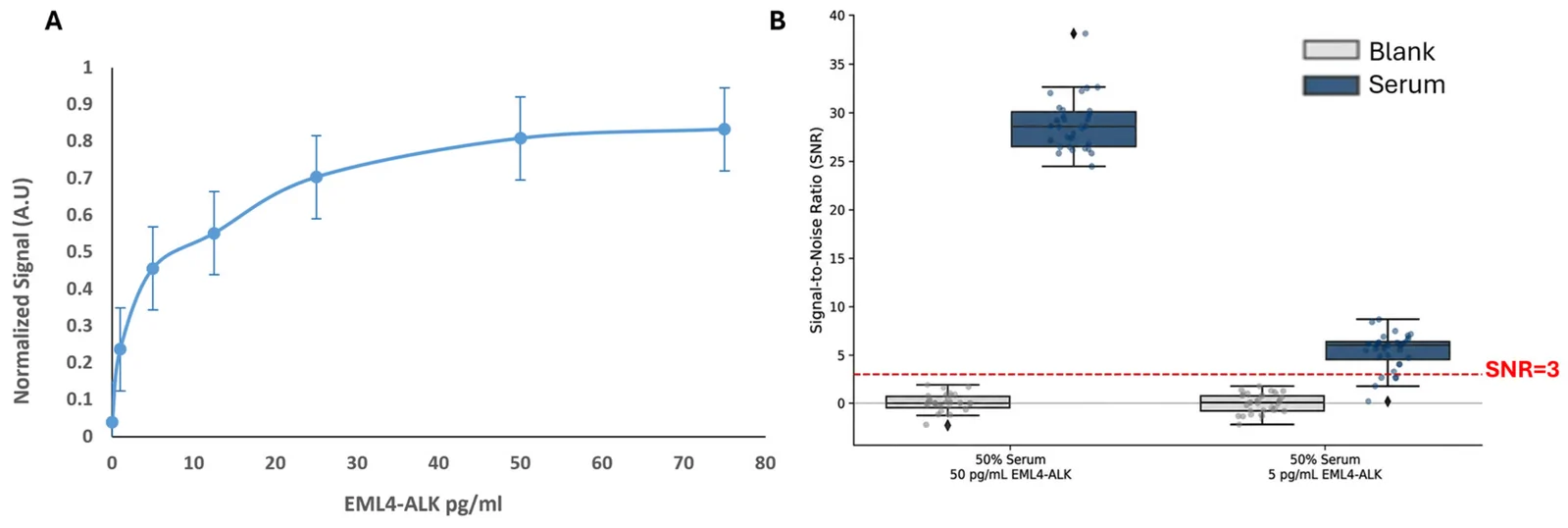
Microfluidic immunoassay sensitivity and matrix tolerance for spiked EML4-ALK in human serum. (**A**) Dose–response of EML4-ALK. Target proteins were captured on-chip using a biotinylated anti-ALK capture antibody and immunodetected using a fluorescently labeled anti-EML4 antibody. Data is normalized between max and min values, and the graph represents aggregated data of 3 experiments (n = 20 each). (**B**) Serum effect on the sensitivity of EML4-ALK. Signal-to-noise ratio (SNR = (Signal − local background)/mean background)) distribution of EML4-ALK fusion protein spiked into 50% human serum at 50 pg/mL (left) and 5 pg/mL (right). Light gray boxes represent blank control samples (50% human serum without target protein); dark blue boxes represent spiked EML4-ALK signal distributions. Overlaid jitter plots display individual replicate measurements (dots) across independent microfluidic detection zones (n = 32 total) from 3 separate experiments. Raw data is presented in Figure S2. The horizontal red dashed line denotes a threshold established at three standard deviations above the blank background mean. Black diamond markers represent statistical outliers. The platform successfully resolves the EML4-ALK fusion protein down to 5 pg/mL directly within complex matrices without pre-analytical purification and only 1:2 sample dilution.

We then evaluated the detection of EML4-ALK inside the microfluidic device. The same anti-EML4-ALK antibody was used in both setups. We used a 4-parameter logistic (4PL) curve to fit the data and extrapolated the sensitivity. A dose–response of EML4-ALK resulted in a limit of blank (LOB = 0.28 pg/mL), limit of detection (LOD = 0.8 pg/mL), limit of quantification (LOQ = 3.5 pg/mL) and CV (%) range of 25–10 in correlation with protein concentration (Figure 3A). To demonstrate the minimal effect of serum, we calculated the SNR for EML4-ALK at two concentrations: one close to the LOQ (5 pg/mL) and one at saturation (50 pg/mL) across two different serum concentrations. The results show that even at 50% serum, we can detect EML4-ALK with an SNR higher than three (Figure 3B). Raw data is available in Figure S2.

**Figure 4:**
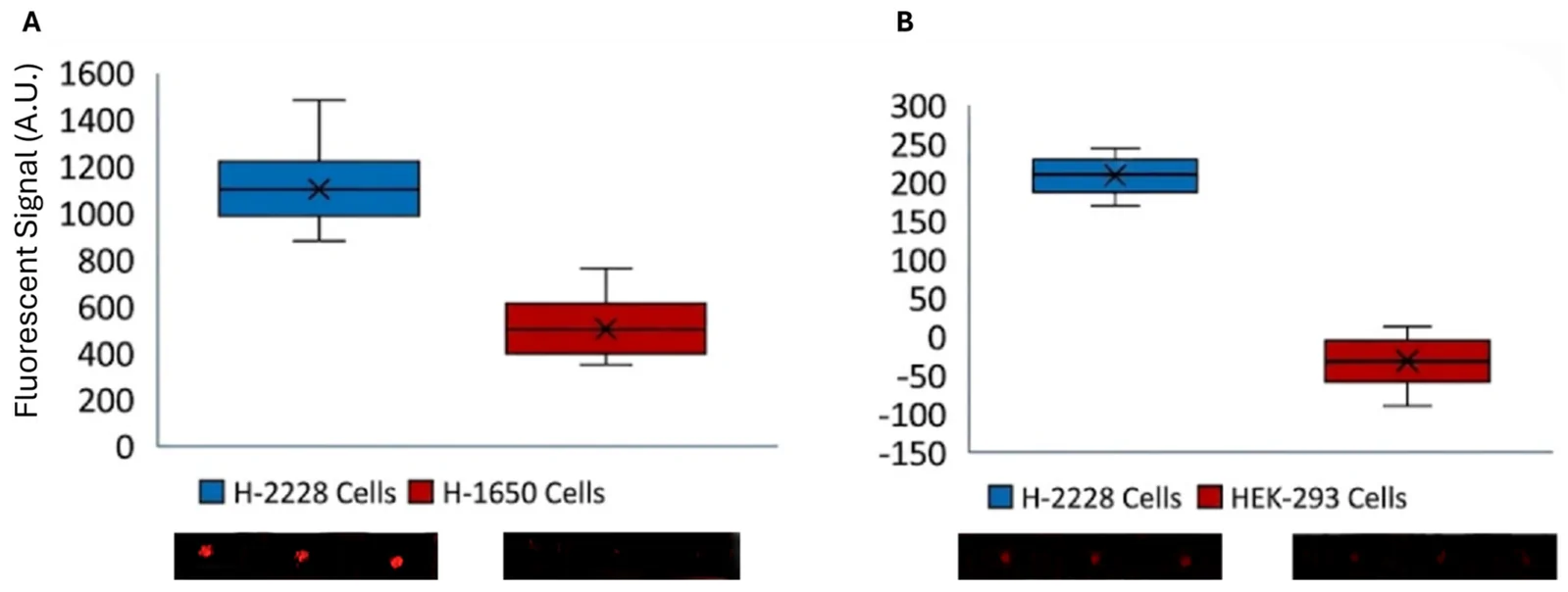
Detection of EML4-ALK in the supernatant of cultivated cell lines. Supernatant was collected from H-2228 and two negative control cell lines, H1650 and HEK-293 cells, cultured in 10 cm dishes. The supernatant was mixed with detergent to release the fusion proteins from exosomes, and the reactions were loaded onto the chip. The fused protein within the samples was captured using a biotinylated anti-ALK antibody and detected using a fluorescently labeled anti-EML4 antibody (n = 20). (**A**, **B**) present the scanned results, showing positive protein capture in the H-2228 cells (red circles) while no protein was detected in the H1650 and HEK-293 cells.

Once the protein was detected in serum, which contains many other biological molecules, we moved towards the detection of EML4-ALK in the supernatant of H-2228 cells. As NSCLC cells secrete ALK via exosomes [15], we expected to detect the mutated fused EML4-ALK protein in the supernatant. The cells were cultured in plates, and the supernatant was collected 48 h after cultivation. A supernatant sample mixed detergent (100 µL total) was flowed through the chip, and the fused protein was immobilized and concentrated on the surface using an anti-ALK biotinylated antibody. Detection was performed using an anti-EML4 fluorescently labeled antibody. As presented in Figure 4, the fused protein was present in the H-2228 cell supernatant but absent in the EGFR H-1650 and HEK-293 negative control groups.

**Figure 5:**
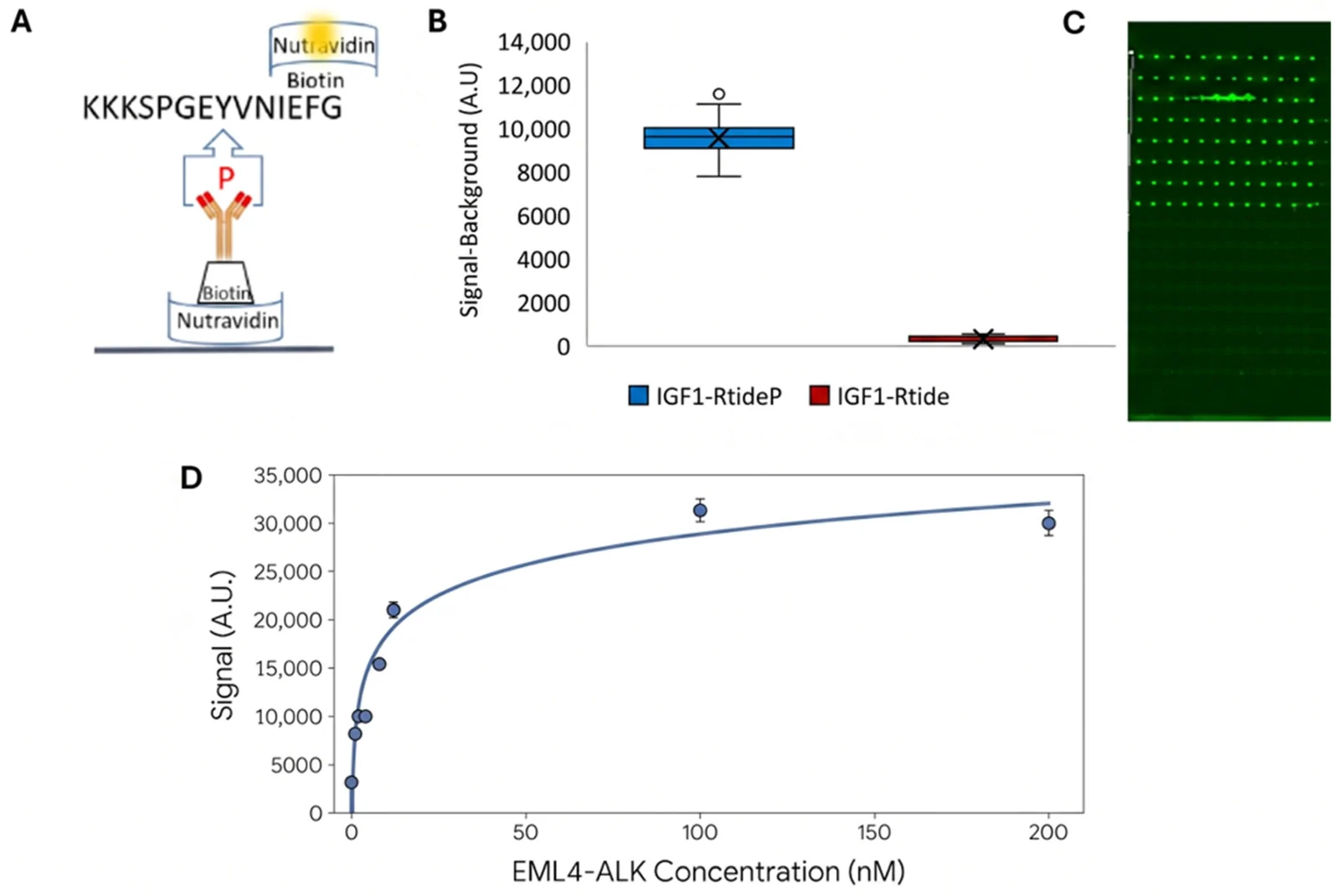
On-chip immobilization and detection of commercial IGF1Rtide-biotin peptide. (**A**) Schematic representation of the experimental setup. The biotinylated peptide was immobilized on the surface using an anti-phospho-tyrosine antibody, and detection was achieved by Cy3-conjugated NeutrAvidin. (**B,C**) On-chip immobilization of phosphorylated IGF1Rtide-biotin peptide versus dephosphorylated peptide. Only the phosphorylated peptide (IGF1-RtideP) was anchored to the chip surface, as indicated by the high signal and fluorescent dots (green) in the scanned image. In contrast, the dephosphorylated peptide (IGF1-Rtide) showed very little binding. (**D**) Calibration of the luminescent ADP-Glo™ Kinase Assay (Promega, Madison, Wisconsin, USA, cat. no. VA7359) with 1 × 10^6^ cells in a plate (n = 3).

Alternatively, because ALK is a kinase, EML4-ALK can be detected using a phosphorylation assay, such as the Kinase Enzyme Systems with ADP-Glo Assay [29]. Here, we compared the detection of EML4-ALK phosphorylation using this kit, with on-chip phosphorylation detection [25,30]. To accomplish this, we used the IGF1Rtide peptide, a 14-amino-acid sequence (KKKSPGEYVNIEFG) derived from the human IRS-1 protein, which was conjugated to biotin. The peptide was immobilized on the device surface using an anti-phospho-tyrosine antibody that binds to phosphorylated tyrosine on the peptide. Detection was then achieved using fluorescently labeled NeutrAvidin (Figure 5A). As shown in Figure 5B,C, the positive phosphorylated peptide was captured on the chip surface, whereas the dephosphorylated peptide was not. The ADP-Glo Assay presented a sensitivity level at the nanomolar scale, like that reported by the manufacturer (Figure 5D), whereas the on-chip assay presented picomolar sensitivity. In other words, the sensitivity was improved by three orders of magnitude compared to that of the conventional assay.

**Figure 6:**
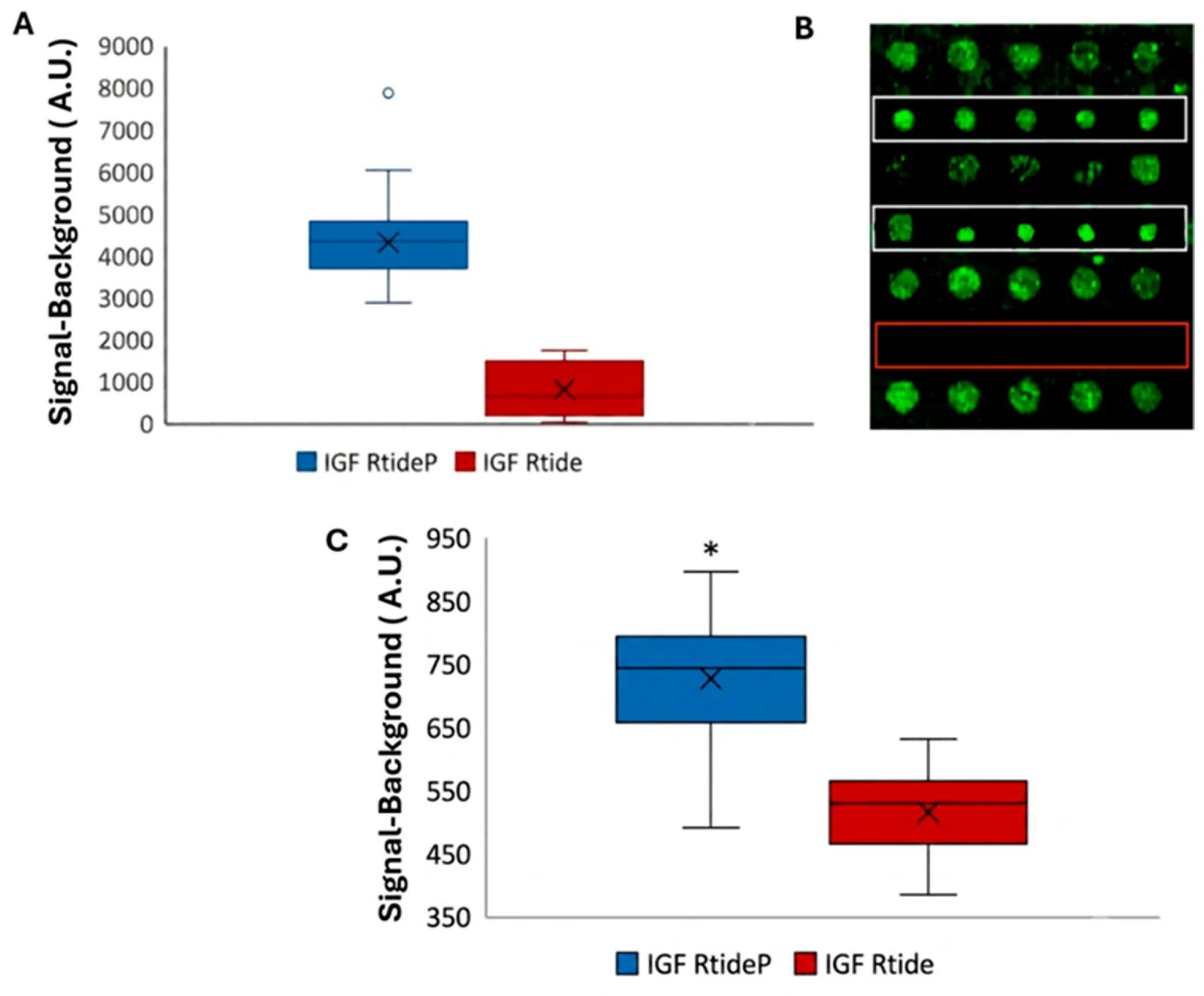
Detection of EML4-ALK kinase activity in cell lysates. IGF1Rtide biotinylated peptide was incubated with homemade H2228 cell lysate in a tube. Then, the sample was flowed in the chip, immobilized via p-Tyr antibody and detected with Cy-3 Streptavidin. (**A**) The signal in the presence of cell extracts was nearly 6-fold higher than in the negative control (in the presence of PBS). (**B**) The high signal yielding from phosphorylated peptide anchored on the buttons area is observed at the 2 upper rows (white rectangles), whereas no signal was observed in the presence of PBS (red rectangular area). The larger green circles are the sample in solution inside the side chambers. (**C**) IGF1Rtide biotinylated peptide was incubated on-chip with homemade H2228 cell lysate and then immobilized via biotin–avidin interaction. Detection of phosphorylated peptide is achieved by fluorescently labeled anti-phospho-tyrosine antibody. T-test (*) *p* < 0.05. n = 32.

Once the experimental setup was optimized, we tested and evaluated the phosphorylation activity in a cell extract model. To do so, phosphorylated and dephosphorylated commercial peptides were incubated with in-house prepared H2228 cell lysates containing the mutant protein (Figure 6). The initial assay included in-tube incubation of the peptide with the H-2228 cell lysate, followed by on-chip immobilization and detection to evaluate the duration required for detectable phosphorylation (Figure 6A,B). The entire experiment was conducted on the chip, including peptide phosphorylation, immobilization, and detection (Figure 6C).

**Figure 7:**
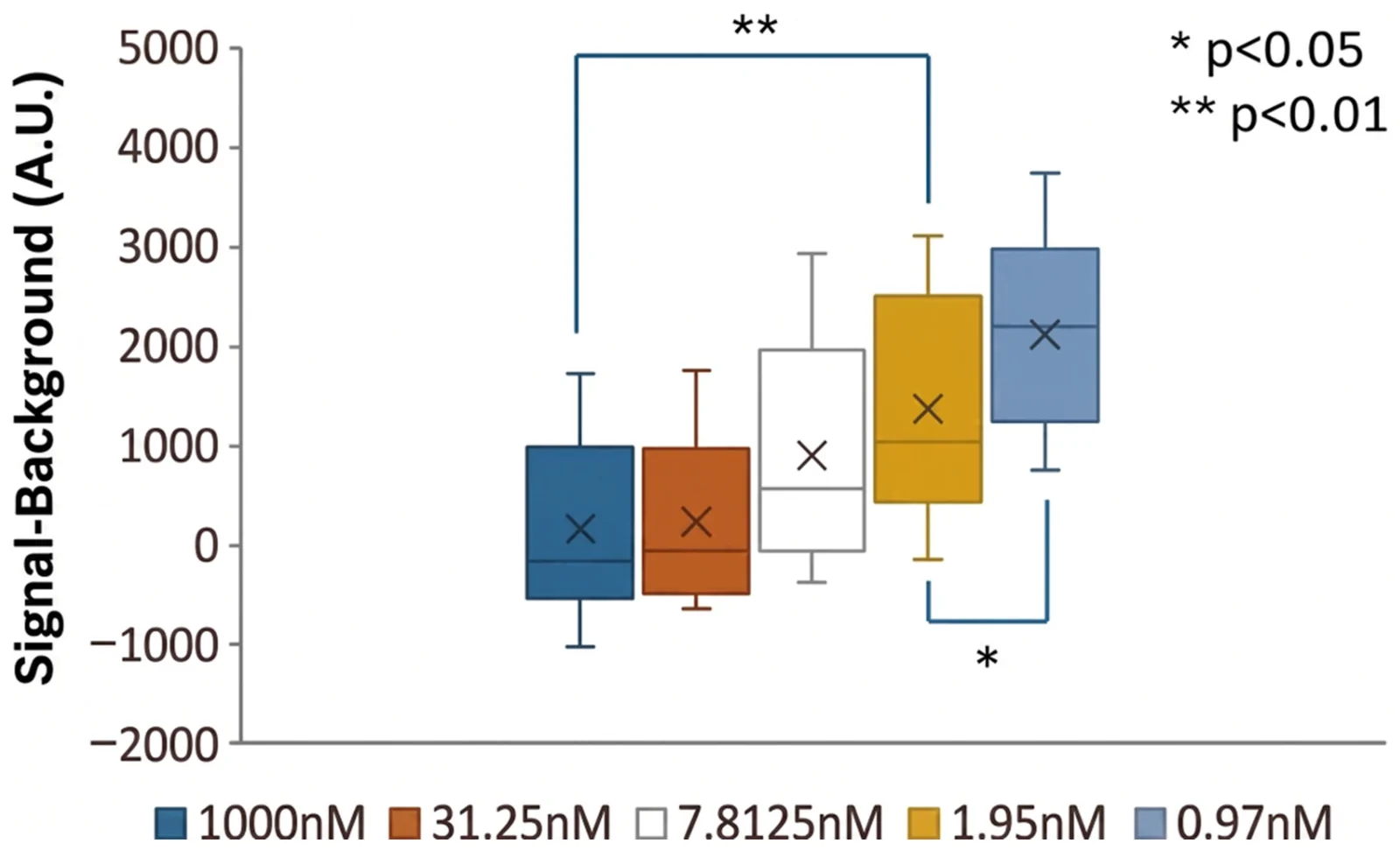
Alectinib inhibition effect on EML4-ALK phosphorylation activity in H2228 cells lysate. H2228 cells lysate was incubated with alectinib (1000 nM–0.97 nM) for 30 min and then incubated with IGF1Rtide-biotin peptide for 30 min. These samples were then applied to the microfluidic experiment (flowed in the device for 30 min). Peptide phosphorylation level was evaluated by a fluorescent antibody (anti-phospho-tyrosine-Alexa 647). A clear correlation between alectinib concentration and the level of phosphorylation inhibition with full inhibition at 31.25 nM was observed vs. minor inhibition at the 0.97 nM concentration.

Our next step was to monitor the impact of therapy on the biomarkers level. We tested the effect of alectinib (MedChem Express HY-13011), a tyrosine kinase inhibitor drug, which was approved as a first-line drug for the treatment of non-small cell lung cancer [31,32] and the evaluation of biomarker level was assessed via the phosphorylation level of IGF1 Rtide peptide, a target of EML4-ALK. Cell lysates were incubated with different concentrations of alectinib (prepared in a final vehicle concentration of 0.002% DMSO) at 37 °C for 30 min, then incubated with the peptide for 30 min, followed by samples flowing within the microfluidic device (at room temperature). As presented in Figure 7, the lowest concentration of alectinib (0.97 nM) induced the minimal decrease in phosphorylation, while higher alectinib concentrations, such as 31.25 nM, almost eliminated EML4-ALK activity. In fact, using 1µM of alectinib induced total inhibition of EML4-ALK kinase activity in the lysate . Applying a concentration of 1.95 nM yielded 50% of the maximal activity (~2000 A.U), which correlates to the IC_50_ level of alectinib (1.9 nM) [33]. Alectinib induced a dose-dependent inhibition of the peptide phosphorylation with full inhibition at the 31.25 nM and minor inhibition at the 0.97 nM concentrations.

## 3. Discussion

The integrated microfluidic platform presented here provides a rapid, cost-effective tool for functional oncological profiling, addressing several critical limitations of currently established methodologies. Crucially, this ultra-sensitive platform offers a distinct translational advantage over standard imaging modalities, whereas radiographic evaluations often exhibit a significant time lag, requiring weeks or months for physical changes in tumor burden to become visible—monitoring circulating protein kinetics can provide near real-time insights into therapeutic efficacy or early disease progression.

Our platform is highly compatible with diverse solid tumors characterized by distinct oncogenic driver mutations, gene fusions, or protein over-expressions. Because of the microscopic fluid volumes managed within the microfluidic channels, the assay requires a sample volume of only 5 µL. This minimal requirement suggests that even small volumes acquired via standard needle biopsies would be sufficient for comprehensive molecular profiling. This diagnostic versatility was validated here against two distinct clinical targets: extracellular VEGF and intracellular EML4-ALK. Notably, the platform successfully captured and quantified EML4-ALK directly from unpurified H-2228 culture supernatants, completely obviating the need for labor-intensive, multi-step exosome isolation, ultracentrifugation, or cell culture extraction protocols. Furthermore, we achieved highly specific detection of the active fusion protein within raw human serum samples following a single, routine centrifugal separation of cellular blood fractions. Similarly, the functional peptide phosphorylation assay required only 5 µL of cell extract, eliminating the need for extensive cell cultivation while facilitating complete process automation.

Although microfluidic architecture offers numerous operational advantages, a primary utility of this specific assay is the integration of standard, commercially available antibodies at sub-microliter reagent volumes. Beyond significantly lowering overall assay costs, this capability directly contributes to the exceptional analytical sensitivity of the platform. Specifically, the integrated MITOMI valve physically isolates and concentrates target molecular complexes directly at the active detection zones, effectively minimizing background noise which is induced from non-specific surface adsorption and substantially lowering the limit of detection.

A notable characteristic of the platform’s performance profile is the compressed dynamic range observed in the low-picogram regime, where the signal approaches early saturation. This behavior is a direct consequence of the exceptionally high spatial density of surface-confined capture sites clustered beneath the actuated MITOMI button valves. With a calculated surface distribution of approximately one antibody per 100 nm^2^ (1 × 10^12^ molecules/cm^2^), the immobilization zone reaches a near-theoretical maximum for molecular packing. This configuration physically isolates and tightly concentrates molecular complexes within a microscopic footprint to drive extreme analytical sensitivity. While the device configuration is fully capable of processing 32 samples in parallel, providing an architectural framework to incorporate automated, on-chip fluidic serial dilutions for the limited clinical scenarios exhibiting high biomarker concentrations, the platform was deliberately optimized for minimal residual disease (MRD) tracking. In the clinical landscape of early-stage detection or post-treatment therapeutic surveillance, circulating target titers are profoundly sparse. Consequently, the analytical capacity to reliably resolve ultra-low concentration thresholds is far more clinically vital than maintaining multi-log upper-bound linearity. By strategically prioritizing a minimized limit of detection over high-concentration dynamic range, this assay delivers maximum diagnostic utility precisely where standard macro-scale modalities encounter their detection limits.

Because low analytical sensitivity remains a primary bottleneck in early oncological therapeutic assessments, implementing multi-modal evaluation strategies is essential. To supplement direct physical quantification of EML4-ALK abundance, we developed a functional kinase assay to monitor its enzymatic tyrosine kinase activity. We quantified the active phosphorylation levels of an ALK-specific peptide substrate on-chip using a fluorescently labeled anti-phospho-tyrosine antibody under both baseline conditions and pharmacological challenge with the selective TKI alectinib. The conventional method for profiling TKI sensitivity involves culturing patient-derived primary cells, exposing them to candidate inhibitors, and measuring ATP consumption using standard commercial assays like ADP-Glo [34] (Figure 5D). However, these multi-well plate assays typically require up to 72 h of incubation, require large starting cell volumes, and are structurally restricted to established, fast-growing commercial cell lines. Establishing patient-derived primary tumor lines for drug screening is a labor-intensive process that can take several months, a delay that is clinically unacceptable for patients requiring immediate therapy selection [35].

Our microfluidic assay offers a rapid, highly sensitive alternative. Following a brief 30 min on-chip incubation of cell lysates with the peptide substrate, target phosphorylation levels were fluorometrically mapped. We observed a clear, dose-dependent correlation between alectinib concentration and EML4-ALK kinase inhibition, achieving complete signaling blockade at 31.25 nM. Crucially, we observed that a therapeutic concentration of 1.95 nM yielded exactly 50% inhibition of maximal kinase activity, showing an outstanding correlation with alectinib’s established cell-free IC_50_ value of 1.9 nM [33]. This high level of physiological accuracy highlights the unique potential of our system as a high-throughput drug-screening platform to evaluate patient-specific responses to single-agent TKIs or combination therapies within hours of biopsy collection [36].

An important limitation of the present study is the absence of matched patient samples collected before and after ALK-directed therapy. Prospective EML4-ALK specimens are difficult to obtain because ALK-rearranged NSCLC represents a relatively small patient subgroup, treatment is often initiated rapidly after molecular diagnosis, and paired pre-treatment/early on-treatment sampling requires close coordination with clinical workflows [37]. We therefore frame the present work as a preclinical feasibility study in recombinant, serum-spiked, and cell-line-derived matrices. In alignment with this early-stage proof of concept, comprehensive analytical standardization, including robust recovery, reagent-lot variation, and formal interference studies, will become a critical milestone for our upcoming clinical validation phases. We anticipate that dissemination of this platform and its analytical performance will help facilitate clinical collaborations and prospective collection of rare ALK-positive samples, enabling future validation against patient response metrics and standard molecular or radiographic readouts.

In conclusion, this integrated microfluidic technology represents a powerful, translational tool for personalized oncology. The platform bears several structural strengths: it minimizes patient discomfort by requiring minimal sample volumes (5 µL), bypasses complex pre-analytical purification workflows, and achieves 15 times higher sensitivity gain over standard plate-based assays via MITOMI-based concentration. By enabling rapid, functional screening of drug candidates directly on primary patient lysates, this platform could assist clinical decision-making, optimize personal therapeutic regimens, and detect early disease recurrence during routine patient follow-up.

## 4. Materials and Methods

### 4.1. Device Fabrication and Interfacing

Microfluidic devices were fabricated using multi-layer soft lithography. Silicon master molds were patterned via maskless photolithography using a Heidelberg MLA 150 maskless aligner (Heidelberg Instruments, Heidelberg, Germany). Replicas were cast using polydimethylsiloxane (PDMS, Sylgard 184, Dow Corning, Midland, MI, USA) mixed at distinct monomer-to-curing-agent ratios to facilitate off-ratio thermal bonding. The control layer mold was cast with a 5:1 (*w*/*w*) PDMS mixture, degassed under vacuum, and partially cured at 80 °C for 30 min. Concurrently, the flow layer mold was prepared by spin-coating a 20:1 (*w*/*w*) PDMS mixture (Laurell Technologies, North Wales, PA, USA) at 2000 rpm for 60 s, followed by a matching partial cure at 80 °C for 30 min. The structured 5:1 control layer was then peeled from its wafer, manually aligned to the spin-coated flow layer under a stereomicroscope, and baked at 80 °C for 1.5 h to achieve monolithic interlayer bonding. The assembled two-layer PDMS chip was subsequently peeled from the flow wafer, punched to create fluidic and pneumatic inlets, and bonded to a clean glass coverslip via oxygen plasma treatment (ambient air, 30% RF power, 30 s). For experimental operation, control and flow lines were interfaced with an external pneumatic manifold using Tygon tubing, enabling automated microvalve execution via custom-programmed software commands.

### 4.2. Surface Functionalization and Localized Antibody Patterning

Surface chemistry functionalization and localized antibody patterning were achieved using an automated, valve-actuated mechanical masking protocol. Initially, biotinylated bovine serum albumin (Biotin-BSA; 1 µg/µL, Thermo Fisher Scientific, Waltham, MA, USA) was perfused through the device for 25 min to establish a baseline biotinylated matrix, followed by the introduction of NeutrAvidin (0.5 µg/µL, Pierce/Thermo Fisher Scientific, Rockford, IL, USA) for 20 min. To spatially pattern the capture regions, the pneumatic button valves were actuated (closed) to physically mask and protect the underlying NeutrAvidin layer within the localized detection zones. The remaining exposed channel architectures were subsequently passivated by perfusing biotinylated polyethylene glycol (Biotin-PEG, 5 kDa, PG2-AMBN-5k, Nanocs Inc., New York, NY, USA;.; 1 µg/µL) for 20 min, preventing non-specific background binding. Following passivation, the button valves were deactivated (opened) to expose the pristine, protected NeutrAvidin surface patches. The target-specific capture interfaces were functionalized by infusing biotinylated antibody, then protein of interest and then, for detection, fluorescently labeled antibody. Between each successive reagent introduction, a PBS wash step to eliminate unbound molecules was applied. For ELISA of VEGF (Figure 2), we aggregated data from 3 separate microfluidic experiments (n = 20 each); the protein used was recombinant Human VEGFA protein (Abcam,Cambridge, UK, cat. no. ab155740). The capture antibody 0.5 ug/mL (Thermos Fisher, Waltham, MA, USA, #P802B) and the detection antibody 90 ng/mL (Thermos Fisher, Waltham, MA, USA, #M808) were fluorescently labeled by Alexa Fluor^®^ 647 Conjugation Kit (Fast)—Lightning-Link^®^ (Abcam, Cambridge, UK, cat. no. ab269823). For microfluidics experiments commercial ALK-EML4 (Abcam, Cambridge, UK, cat. no. ab268523) was used as the protein source, while capturing biotinylated anti-ALK mAB (Cell Signaling Technology, Danvers, MA, USA, cat. no. #8936, lot 1) was used and the detection anti-EML4 mAB (Cell Signaling Technology, Danvers, MA, USA, cat. no. #12156, lot 1). For lysate cells experiments 0.2 µg/µL biotinylated anti-EML4-ALK antibody (Abcam, Cambridge, UK, cat. no. ab6658) or 0.01 µg/µL biotinylated anti-pTyr antibody (Cell Signaling Technology, Danvers, MA, USA, cat. no. 2908S) were flowed for 20 min. The channels were thoroughly rinsed with phosphate-buffered saline (PBS, pH 7.4).

### 4.3. On-Chip Assay Execution and Target Detection Protocol

Following surface passivation and mechanical matrix patterning, the active microfluidic assay was executed using a standardized sample infusion and target labeling sequence. For each diagnostic run, a 5 µL volume of the target biological sample, consisting of either recombinant protein spiked into crude human serum, harvested cell culture supernatant, or whole-cell lysates subjected to varying pharmacological drug challenges (e.g., alectinib), was diluted in an equal volume (5 µL) of a specialized processing buffer fortified with mild detergents (e.g., 0.5% *v*/*v* Nonidet P-40). This detergent-mediated dilution step was utilized to systematically disrupt extracellular vesicle membranes (exosomes), thereby fully liberating encapsulated intracellular drivers like the EML4-ALK fusion protein for surface-immobilized antibody engagement. The resulting 10 µL sample mixture was continuously perfused through the microfluidic flow channels at a volumetric flow rate of 1 µL/min for a total duration of 10 min to optimize convective transport dynamics and maximize capture efficiency within the localized, valve-protected detection zones. Following complete sample delivery, the channel architectures were flushed with a continuous stream of phosphate-buffered saline (PBS, pH 7.4) to rapidly evacuate residual complex matrices, unlysed debris, and non-specifically adsorbed background components. The physically trapped molecular complexes were subsequently labeled on-chip by infusing a secondary primary or secondary fluorescently conjugated detection antibody (e.g., anti-EML4-Alexa 647 or anti-phospho-tyrosine-Alexa 647) at target working concentrations for an incubation period of 20 min. Unbound labeling agents were cleared via a final, high-stringency fluidic rinse cycle with PBS. To maintain molecular stability and prevent signal degradation, the microfluidic control channels were held under constant pneumatic actuation to lock the target configurations beneath the button valves. The fully processed microfluidic chip was then immediately transitioned to an LS Reloaded microarray laser scanner (Tecan) for automated fluorometric screening and regional signal processing, as detailed in Section 4.7.

### 4.4. Cell Line Maintenance and Culture Conditions

The human non-small cell lung cancer (NSCLC) cell lines NCI-H2228 (EML4-ALK positive; ATCC Catalog Number: CRL-5935) and NCI-H1650 (EGFR mutant; ATCC Catalog Number: CRL-5883) were maintained as adherent monolayers in 10 cm tissue culture-treated dishes (Lumitron). Cells were cultivated in RPMI 1640 medium supplemented with 10% fetal bovine serum (FBS), 1% L-glutamine, and 1% penicillin–streptomycin (all culture reagents sourced from Biological Industries, Beit HaEmek, Israel). Cultures were maintained at 37 °C in a humidified atmosphere containing 5% CO2 using a Galaxy 170 S incubator (New Brunswick, Edison, NJ, USA). Cells were routinely passaged upon reaching 80–90% confluence using standard trypsinization protocols. To maintain genetic stability and phenotypic consistency, cells were kept in continuous culture for a maximum of 15 passages before being discarded and replaced from frozen stocks.

### 4.5. Western Blot Analysis

Protein samples were mixed with 4× Laemmli sample buffer and denatured at 96 °C for 10 min. The denatured samples were resolved via sodium dodecyl sulfate-polyacrylamide gel electrophoresis (SDS-PAGE) on freshly prepared 10% polyacrylamide resolving gels using a standard Tris-glycine running buffer. Following separation, the proteins were electro-transferred onto a nitrocellulose membrane (0.2 µm pore size, Bio-Rad Laboratories, Hercules, CA, USA,, cat. no. 162-0115) using a Trans-Blot Turbo Transfer System (Bio-Rad Laboratories, Hercules, CA, USA,). Membrane transfer quality and total protein loading uniformity were visually verified by staining with Ponceau S solution (Sigma-Aldrich, St. Louis, MO, USA, cat. no. 81462). Membranes were subsequently rinsed with Tris-buffered saline (TBS) to remove the stain, blocked with 5% (*w*/*v*) skimmed milk powder in TBS containing 0.1% Tween-20 (TBST) for 1 h at room temperature, and washed. The membranes were then incubated with a primary antibody solution containing biotinylated anti-EML4-ALK antibody (prepared in PBS supplemented with 2.5% *w*/*v* bovine serum albumin and 0.05% *w*/*v* sodium azide). Fluorescent target detection was achieved via subsequent incubation with a NeutrAvidin-Cy3 (NA-Cy3) conjugate, allowing precise visualization of the bound biotinylated antibody complexes.

### 4.6. Cell Lysate Preparation

Prior to harvesting, cells were rinsed twice with ice-cold phosphate-buffered saline (PBS). Cellular lysis was performed by incubating the monolayers on ice for 30 min in an ice-cold lysis buffer composed of 50 mM Tris (pH 7.6), 150 mM NaCl, 5 mM EDTA (pH 8.0), and 0.5% (*v*/*v*) Nonidet P-40 (NP-40). The buffer was freshly supplemented with a complete protease inhibitor cocktail (Roche, Basel, Switzerland, cat. no. 4693159001), 1 mM phenylmethylsulfonyl fluoride (PMSF), and phosphatase inhibitor cocktails (Sigma-Aldrich, St. Louis, MO, USA, cat. nos. P5726 and P0044). To preserve protein phosphorylation states, the lysis architecture was further fortified with the following endogenous phosphatase inhibitors: 10 mM NaF, 20 mM β-glycerophosphate, 1 mM sodium orthovanadate (Na_3_VO_4_), and 20 mM *p*-nitrophenyl phosphate. Following the 30 min incubation on ice, insoluble cellular debris was pelleted via centrifugation at 14,000× *g* for 45 min at 4 °C. The resulting clarified supernatant containing the whole-cell lysate was collected, quantified, and stored at −80 °C until microfluidic chip infusion.

### 4.7. Fluorescence Image Acquisition and Signal Quantification

Microfluidic chips were scanned using an LS Reloaded microarray laser scanner (Tecan, Männedorf, Switzerland), and initial image data extraction was performed using GenePix Pro 7.0 software (Molecular Devices, San Jose, CA, USA). Downstream image visualization, preparation of figures, and secondary array analysis were executed using ImageJ software 1.54j (NIH, Bethesda, MD, USA). To account for baseline fluorescence variations arising from minor non-specific antibody adsorption to the passivated channel walls, a localized background correction protocol was applied. The specific protein capture signal was quantified as the mean fluorescence intensity localized directly beneath the deactivated button valve geometry. Local background values were calculated within an annular region centered on each individual button feature, spanning an outer boundary radius of 2R (where R represents the primary valve radius) with a 2-pixel offset spacing from the valve perimeter to isolate edge reflection and boundary scattering artifacts. The final corrected fluorescence intensity for each technical replicate was determined by subtracting this local localized annular background value from the raw internal feature signal. We used analytical method validation according to CLSI EP17-A2/IUPAC guidelines.

Determination of Detection Capabilities (LOB, LOD, and LOQ) were calculated as follows:

(a) The blank distribution was established by measuring n independent baseline zones. Mean intensity and standard deviation of the blank were utilized to define the analytical limits (LOB, LOD, and LOQ).
(b) LOB = Mean Blank + 1.645 × S.D. (Blank), LOD (signal) = LOB + 1.645 × S.D. (The lowest concentration signal), LOQ = Mean Blank + 10 × S.D. (Blank).
(c) Acceptance Criteria: The calibration runs were accepted based on predefined analytical criteria:
  (i) Goodness of fitting in the 4-parameter logistic (4PL) fitting curve for both experiments was $R^{2}>0.99$.
  (ii) Replicate precision with coefficient of variation CV (%) (σ-S.D., μ-mean).
    $$CV=\left(\frac{\sigma }{\mu }\right)\times 100$$
  (iii) The signal-to-noise ratio SNR (μ-mean, σ-S.D.).
    $$SNR=\frac{\mu_{\mathrm{signal}}-\mu_{\mathrm{blank}}}{\sigma_{\mathrm{blank}}}$$
(d) Fitting method—The 4-parameter logistic (4PL) fitting curve regression equation:
  $$Y=d+\frac{a-d}{1+{\left(\frac{X}{c}\right)}^{b}}$$
  X = Protein concentration (pg/mL), Y = Fluorescent signal, a = Minimum asymptote (background signal), d = Maximum asymptote (saturation signal), c = Inflection point (concentration at 50% maximal response), and b = Hill slope (steepness factor of the curve).

Using the slope, we were able to calculate the LOD concentration which indicates the method sensitivity by determining.

How much the signal jumps for every 1 pg/mL of protein added:

$$LOD_{\mathrm{concentration}}=\frac{3.3\times \sigma_{\mathrm{blank}}}{\mathrm{Slope}}$$

### 4.8. EML4-ALK Kinase Activity Quantification via ADP-Glo™ Assay

Enzymatic activity of the EML4-ALK kinase system was evaluated using the luminescent ADP-Glo™ Kinase Assay (Promega, Madison, WI, USA, cat. no. VA7359). This homogeneous assay format quantifies kinase-mediated catalysis by monitoring the accumulation of adenosine diphosphate (ADP) produced during the kinase reaction, wherein the enzyme utilizes adenosine triphosphate (ATP) as a phosphoryl donor. Following the primary enzymatic incubation, the unreacted ATP fraction was depleted. The accumulated ADP byproduct was subsequently converted back into ATP via the addition of the Kinase Detection Reagent. This newly synthesized ATP pool was utilized in a coupled luciferase reaction to catalyze the conversion of luciferin into oxyluciferin, generating a stable bioluminescent signal directly proportional to the concentration of generated ADP. Luminescence was recorded using a microplate luminometer (plate reader), and absolute kinase activity was quantified by interpolating raw luminescent values against a prepared ATP-to-ADP standard conversion curve.

## Figures and Tables

**Table 1:** Comprehensive statistical analysis comparing ELISA on-chip to standard ELISA. Precision/CV% was provided for the lowest concentration.

Parameter	On-Chip	Standard
LOD_Concentration_	1.5 pg/mL	23.3 pg/mL
LOQ	5.1 pg/mL	39.8 pg/mL
Slope (S)	0.0125	0.00257
Precision/CV%	3.12% (at 3.9 pg/mL)	26.12% (at 31.25 pg/mL)

## Data Availability

Data is accessible through a link in the supplementary information.

## Supplementary Materials

The following supporting information can be downloaded at: https://www.mdpi.com/article/10.3390/bios16090484/s1. Figure S1. Biotinylated phosphorylated peptide (commercial) immobilization on–Chip by biotin and detection with anti–Phospho–Tyrosine Alexa 647. A. For on–chip detection of phosphorylated peptides two protocols were applied: Blue—the peptide flowed within the chip, allowing it to bind via Avidin–Biotin interaction. Orange—The chip tubes were filled with the peptide and remained for an incubation period of 20 min allowing it to bind to the surface. Then, anti-phospho-Tyrosine Alexa 647, was applied to allow detection of the phosphorylated peptide. Signal levels (Mean ± S.D.) were normalized to the maximum value. B. Real time screen of the signals within the microfluidic chip. The small dots are the buttons valves in which the protein is trapped—the difference in signal intensity results from different concentrations of the peptide. The large circles are the side chambers in which the peptide is in a solution state—without anchoring. Flowing the peptide yielded a ten–fold higher level of signal versus peptide incubation and concentrating of the peptide from miniscule sample amounts. Figure S2. Evaluation of EML4–ALK fusion protein assay sensitivity and matrix tolerance across varying serum concentrations. The multi–panel plot demonstrates the specific and robust discrimination of the EML4–ALK fusion protein from complex biological matrices using three independent detection antibody setups. (Left) 10% Serum Matrix: Detection of 5 pg/mL EML4–ALK (N = 20). (Middle) 50% Serum Matrix: Detection of a 50 pg/mL target spike within a high–density matrix (N = 30). (Right) 50% Serum Matrix (High Sensitivity): Resolution of a minimal 5 pg/mL target concentration inside a dense 50% serum background (N = 30). Box boundaries define the interquartile. range (IQR) from the 25th to the 75th percentile; internal horizontal lines represent the median value. Whiskers extend to 1.5 \times IQR, diamonds denote statistical outliers, and individual technical replicates are overlaid as semi–transparent jittered points to display the complete distribution of the raw data. Below the right panel, we show an example of fluorescent spots taken from the scanner. Fluorescence images, scanned at 633 nm wavelength, were processed using a spatial circular mask to isolate representative raw signals of the on–chip experiment. Image shows a duplicate of EML4–ALK (5pg/ml) in 50% serum (top) compared with a duplicate of 50% serum only marked as Blank (bottom). Figure S3. Western blot simulating EML4–ALK standard limit of detection using the same antibodies used for the on–chip experiments. A range of 0.005–50ng/ml EML4–ALK was uploaded in lane 1–4, and the ladder was applied on lane 5. The EML4–ALK band was observed starting from concentrations of 5ng/ml protein.
