# Supplementary material for: Monitoring EML4-ALK Biomarkers via a Microfluidic Assay for Treatment Efficacy Assessment
Source: Biosensors (Basel). 2026 Sep 2;16(9):484. doi: 10.3390/bios16090484 (PMC13604303; doi:10.3390/bios16090484)
Supplement: Supplementary file 1 [file biosensors-16-00484-s001.zip › biosensors-4426846-supplementary.pdf]

Monitoring EML4-ALK Biomarkers via a Microfluidic Assay for Treatment Efficacy Assessment

*Shaked Doron, *Lev Brio, *Matan Krasner, Efrat Barbiro-Michaely, **Doron Gerber

The Mina and Everard Goodman Faculty of Life Sciences and the Institute for Nanotechnology and Advanced Materials, Bar Ilan University, Ramat-Gan, Israel.

*Equally contributing authors

** Corresponding author

Corresponding author doron.gerber@biu.ac.il

Keywords: Integrated microfluidics, minimal residual disease, NSCLC, anaplastic lymphoma kinase, CTCs

Supplementary Figures

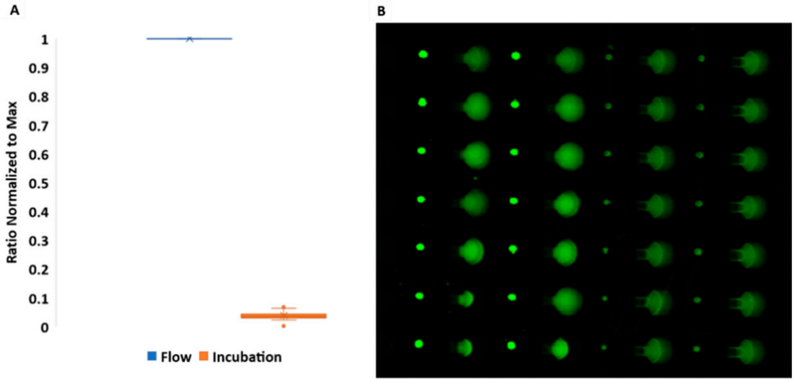

Figure S1. Biotinylated phosphorylated peptide (commercial) immobilization on-Chip by biotin and detection with anti-Phospho-Tyrosine Alexa 647. **A.** For on-chip detection of phosphorylated peptides two protocols were applied: **Blue -** the peptide flowed within the chip, allowing it to bind via Avidin-Biotin interaction. **Orange -** The chip tubes were filled with the peptide and remained for an **incubation** period of 20 min allowing it to bind to the surface. Then, anti-phospho-Tyrosine Alexa 647, was applied to allow detection of the phosphorylated peptide. Signal levels (Mean ± S.D.) were normalized to the maximum value. **B. Real time screen of the signals within the microfluidic chip.** The small dots are the buttons valves in which the protein is trapped – the difference in signal intensity results from different concentrations of the peptide. The large circles are the side chambers in which the peptide is in a solution state – without anchoring. Flowing the peptide yielded a ten-fold higher level of signal versus peptide incubation and concentrating of the peptide from miniscule sample amounts.

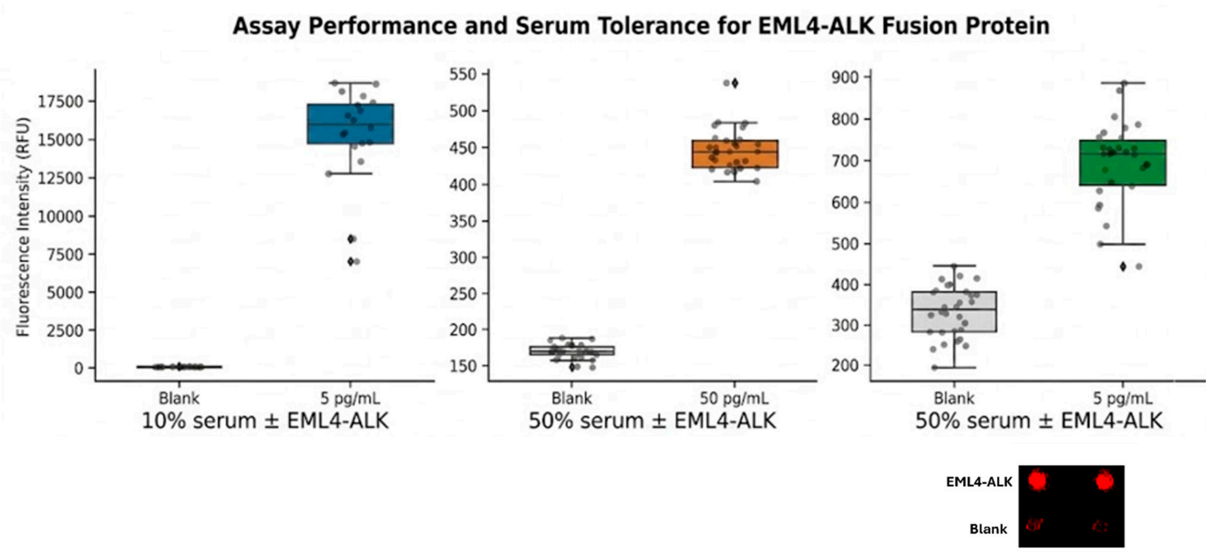

Figure S2. Evaluation of EML4-ALK fusion protein assay sensitivity and matrix tolerance across varying serum concentrations. The multi-panel plot demonstrates the specific and robust discrimination of the EML4-ALK fusion protein from complex biological matrices using three independent detection antibody setups. **(Left) 10% Serum Matrix:** Detection of **5 pg/mL** EML4-ALK (N = 20). **(Middle) 50% Serum Matrix:** Detection of a **50 pg/mL** target spike within a high-density matrix (N = 30). **(Right) 50% Serum Matrix (High Sensitivity):** Resolution of a minimal **5 pg/mL** target concentration inside a dense **50% serum** background (N = 30). Box boundaries define the interquartile. range (IQR) from the 25th to the 75th percentile; internal horizontal lines represent the median value. Whiskers extend to 1.5 \times IQR, diamonds denote statistical outliers, and individual technical replicates are overlaid as semi-transparent jittered points to display the complete distribution of the raw data. Below the right panel, we show an example of fluorescent spots taken from the scanner. Fluorescence images, scanned at 633 nm wavelength, were processed using a spatial circular mask to isolate representative raw signals of the on-chip experiment. Image shows a duplicate of EML4-ALK (5pg/ml) in 50% serum (top) compared with a duplicate of 50% serum only marked as Blank (bottom).

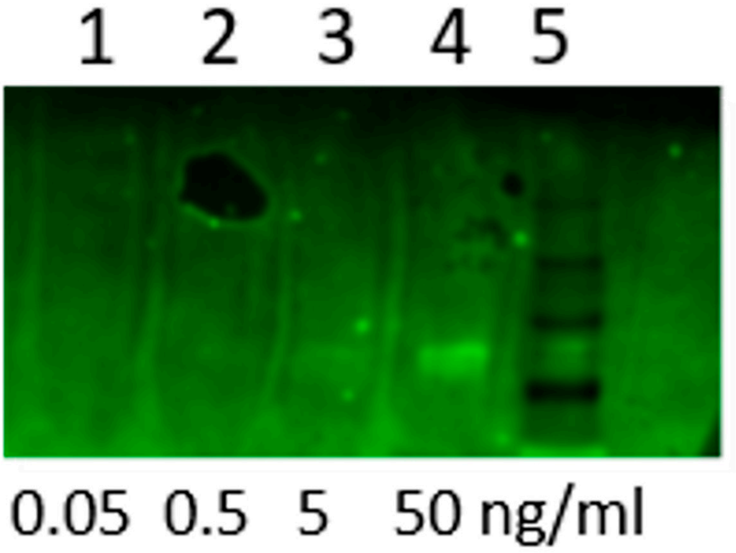

Figure S3. Western blot simulating EML4-ALK standard limit of detection using the same antibodies used for the on-chip experiments. A range of 0.005-50ng/ml EML4-ALK was uploaded in lane 1-4, and the ladder was applied on lane 5. The EML4-ALK band was observed starting from concentrations of 5ng/ml protein.

Raw data is available for download in the following link:
"https://www.dropbox.com/scl/fo/aqopjrlw6npx1cqf4ar5l/AOskjqK_EbmhJqHdWKZJaPw?rlkey=f9cv69mlhvv5u942klnc5dwym&dl=0"
